# The Divorce between Medicine and Surgery

**Published:** 1894-12-08

**Authors:** 


### The Hospital

An Institutional Journal of

The Medical Sciences and Hospital Administration, with

AN EXTRA NURSING SUPPLEMENT.

Vol. XVII.—No. 428.] [Dec. 8, 1894.

### The Divorce Between Medicine and Surgery.

In few thing does the artificial and abrupt division between medicine and surgery, which has been handed down to us by our forefathers, show to such disadvantage as in the text-books which are prepared by professors of medicine. In the daily work of life it is the individual patient which has to be studied, and in so doing every fact bearing on the case is taken into consideration, whether it touches the medical or the surgical side. Quite different is it in the text-books where a one-sided view is too often taken, the physician considering only what he can do by his pills and the surgeon by his operations. To the student the result is confusing and embarrassing in the last degree. He expects, under the head of treatment. to get not merely a list of possible remedies but some guidance as to the sort of case in which each may be most usefully employed, and as regards drugs this information is often given. But in regard to the grave question, which every year is becoming a larger and larger one, whether and when drugs must be laid aside and recourse be had to surgical interference, medical books are distressingly silent. The method of interference is a surgical question, but, unless we are boldly to accept the ridiculous proposition that a surgeon is only to be called in as a last resource, the knowledge of the particular conditions which are capable of relief by surgery, and therefore demand surgical treatment, are surely as important branches of medical knowledge as are the indications for acids or alkalis, for calomel or iodide of potassium.

As an example of the incompleteness of what one may call the medical view we may instance a very popular text-book, of which a new edition has just been published, which in dealing with the wide and far-reaching question of peritonitis is contented to say, "The questions relating to *operative procedures* in cases of acute peritonitis have now come into conspicuous prominence. They belong, however, entirely to the domain of surgery, and for most valuable information on this part of the subject reference may be made to Mr. Treve's lectures." That is all the hint that is given to the student in reference to the enormously important subject of operative interference in peritonitis, except that in another paragraph one of "the main indications" for treatment is stated to be "to have recourse to operative measures in appropriate cases." But what are appropriate cases, and when is the surgeon to be called in? These questions are not answered, and not the slightest hint is given as a guide to practice. If a physician writing a book like this chose to say that every case of peritonitis should be handed over to the surgeon, things would be simplified; but when he deals with the disease as within his province and gives, under the head of treatment, rest, diet, removal of blood, hypodermic morphia, quinine, and local applications, poultices, &c., while, as regards surgical interference, upon the early recourse to which in so many cases the life of the patient hinges, he says nothing more than is given above, we cannot but feel that the view taken of the case is one-sided and delusive, and is in no sense a proper one to teach to students.

*Mutatis mutandis*, almost the same criticism may be applied to the chapter on intestinal obstruction in the same work. Obstructions from all causes are lumped together, and a list of operations which can be performed is given, but no attempt is made to indicate the sort of cases in which they may be usefully employed. Truly it is said that intestinal obstruction belongs chiefly to the domain of surgery, but it is nowhere said that every case is surgical, nor are such details given as will enable the practitioner to make a selection. Again, as regards gall-stones and affections of the gall bladder, operations are mentioned, but we are left in the dark as to the circumstances under which they may properly be performed.

The one-sidedness of what one may call the physician's view is curiously illustrated again in regard to the treatment of acute cerebral inflammations. As every practitioner knows there is one particular form, that arising from disease of the temporal bone, which is both peculiarly fatal and peculiarly within the reach of surgical treatment. Under the head of diagnosis details are given drawing attention to this connection, but under treatment nothing is said except that where an abscess can be definitely localised in the cortex surgical treatment should be had recourse to; no hint is given that cerebral symptoms in ear disease stand apart from others forms and should be surgically considered from the first.

From the medical point of view no doubt students are well taught by physicians, but we cannot look upon it as satisfactory that physicians should fail to define the point at which cases apparently medical become surgical. It is no use saying in a general sort of way that "this aspect of the subject belongs to the domain of surgery." If diseases which are apt become surgical are treated of at all in a book on medicine they should be so dealt with as at least to show when the cases should be considered from a surgical point of view. A book on medicine written by a surgeon would be an interesting work, and would form a far thicker volume than many people imagine.

